# Management of locally advanced lynch syndrome rectal cancer during pregnancy with neoadjuvant immunochemotherapy: a case report

**DOI:** 10.3389/fonc.2026.1813750

**Published:** 2026-05-20

**Authors:** Shaoqing Fan, Xiurong Li, Li Shi, Jianan Dong

**Affiliations:** 1 Department of General Surgery, The Fourth Hospital of Hebei Medical University, Shijiazhuang, Hebei, China; 2 Department of Obstetrics and Gynecology, The Fourth Hospital of Hebei Medical University, Shijiazhuang, Hebei, China; 3 Department of Obstetrics and Gynecology, The Second Hospital of Hebei Medical University, Shijiazhuang, Hebei, China

**Keywords:** Immunotherapy, lynch syndrome, neoadjuvant chemoradiotherapy, pregnancy, rectal cancer

## Abstract

**Background:**

Lynch syndrome (LS) is a hereditary cancer predisposition syndrome. Colorectal cancer during pregnancy is extremely rare, and LS-associated cases pose unique diagnostic and therapeutic challenges due to maternal-fetal and genetic considerations.

**Case presentation:**

A 37-year-old woman at 22 + 5 weeks of gestation presented with hematochezia. Ultrasonography and pelvic MRI revealed a 7.2 × 6.3 × 6.1 cm rectal mass with multiple enlarged pelvic lymph nodes posterior to the gravid uterus. Colonoscopy confirmed an ulcerative lesion 10 cm from the anal verge. Histopathology and immunohistochemistry (MLH1-/PMS2-/MSH2+/MSH6+) confirmed LS-associated rectal adenocarcinoma. Given the poor fetal viability and potential teratogenic effects of systemic therapy, medical termination was performed. The patient subsequently underwent neoadjuvant chemoradiotherapy (50.4 Gy in 28 fractions) combined with two cycles of capecitabine plus oxaliplatin (CapeOx) and tislelizumab, achieving significant tumor regression (yT2N0M0). Laparoscopic Dixon surgery revealed a 2.5 × 1.5 × 1.0 cm ulcerative lesion, corresponding to Tumor Regression Grade (TRG) 1. Six cycles of adjuvant CapeOx with tislelizumab were completed. No severe adverse events occurred, and at 24 months follow-up, the patient remains disease-free.

**Conclusion:**

LS-associated rectal cancer during pregnancy requires individualized, multidisciplinary management. Medical termination followed by neoadjuvant immunochemoradiotherapy can optimize maternal outcomes while minimizing fetal and genetic risks.

## Case description

A 37-year-old woman (gravida 4, para 2) presented at 22 + 5 weeks gestation with a 1-month history of hematochezia. She had no significant past medical history. Family history was notable for a father diagnosed with Lynch syndrome-associated colorectal cancer. Physical examination revealed BMI 24.4 kg/m², fundal height of 16 cm, abdominal circumference 91 cm, non-engaged fetus, and fetal heart rate 150 bpm.

**Figure 1:**
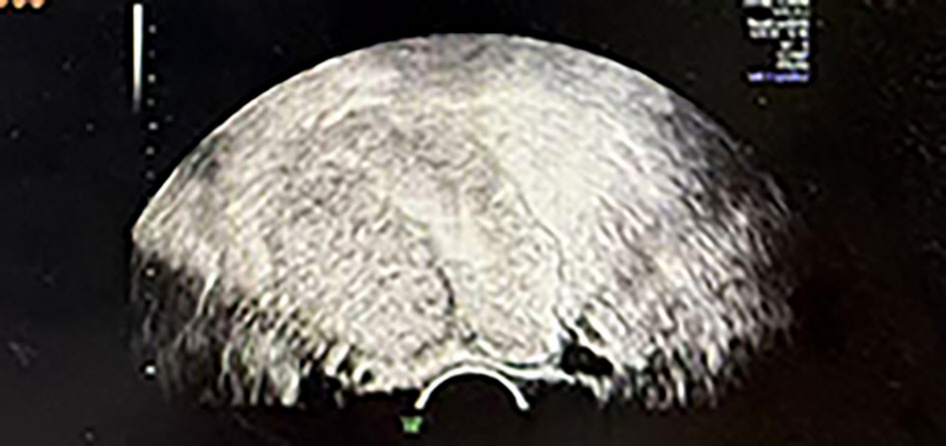
Obstetric ultrasound examination. Ultrasonography revealed a solid pelvic mass measuring approximately 6.8 × 6.3 cm located posterior to the gravid uterus.

**Figure 2:**
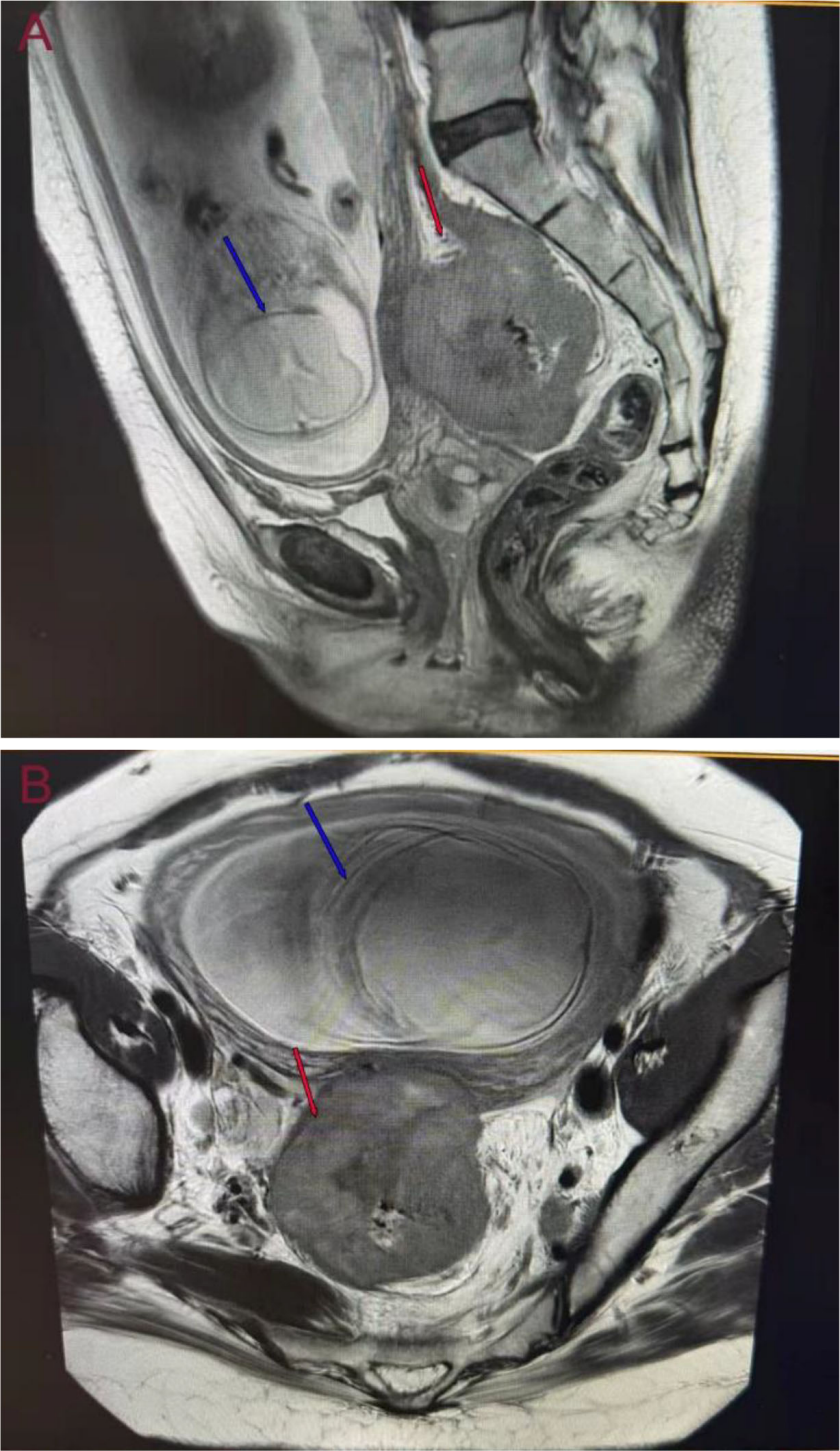
Pelvic MRI findings. **(a)** sagittal and **(b)** coronal views showing a rectal mass (red arrow) located posterior to the gravid uterus (blue arrow, fetus). The lesion measured approximately 7.2 × 6.3 × 6.1 cm and was accompanied by multiple enlarged pelvic lymph nodes(cT4N2M0).

**Figure 3:**
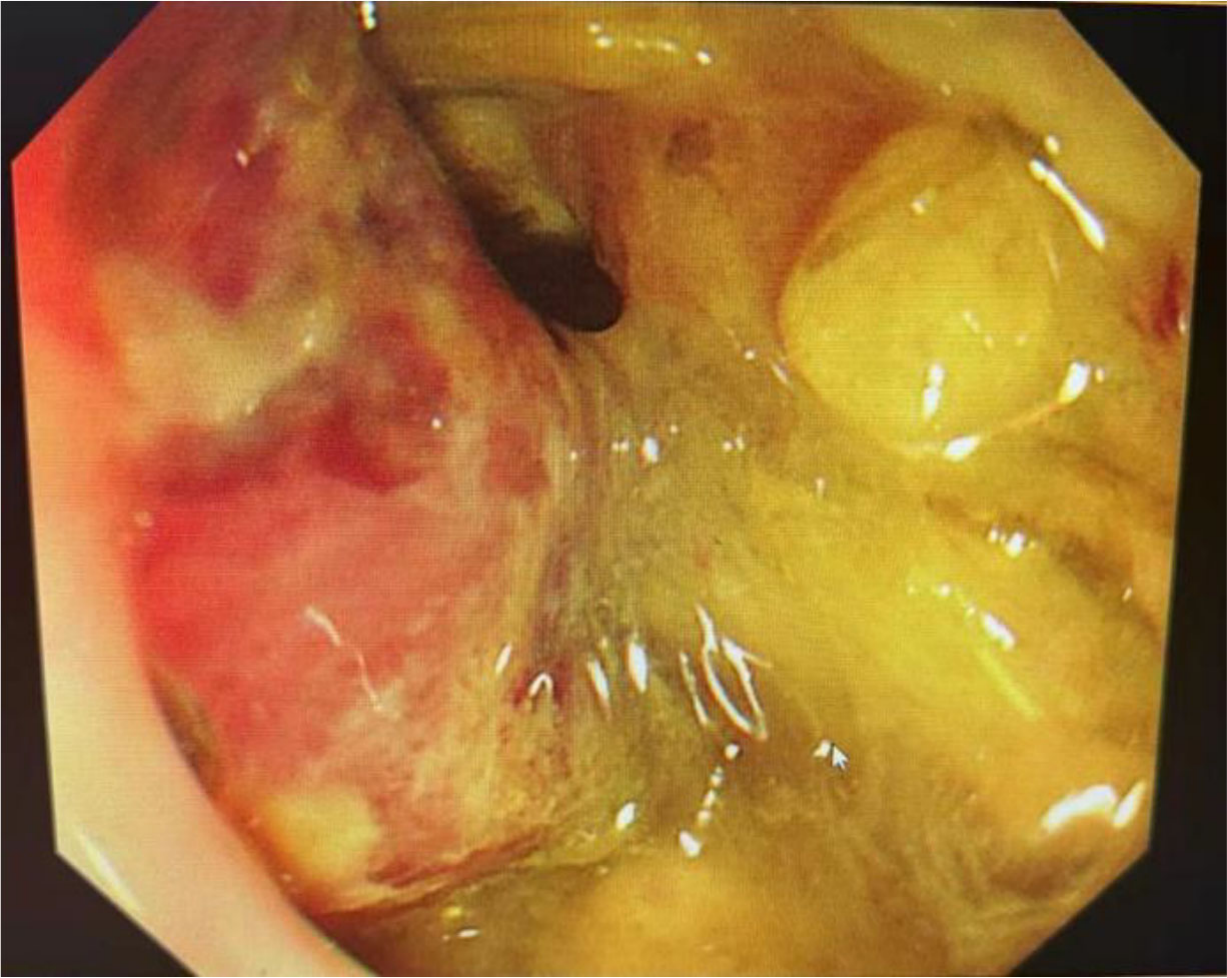
Colonoscopy findings. Endoscopic view showing an ulcerated rectal lesion approximately 10 cm from the anal verge, nearly obstructing the lumen.

Obstetric ultrasound revealed a solid pelvic mass measuring 6.8 × 6.3 cm posterior to the gravid uterus (**Figure 1**). Pelvic MRI confirmed a rectal mass measuring 7.2 × 6.3 × 6.1 cm with multiple enlarged pelvic lymph nodes, posterior to the uterus (**Figures 2a, b**, cT4N2M0). Colonoscopy identified an ulcerative lesion 10 cm from the anal verge, nearly obstructing the lumen (**Figure 3**). Chest and abdominal CT showed no distant metastases. Laboratory tests revealed normal CEA (1.28 ng/mL), elevated CA 19-9 (232.7 U/mL), and elevated AFP (186.1 ng/mL). Histopathology confirmed adenocarcinoma; immunohistochemistry showed MLH1-/PMS2-/MSH2+/MSH6+, consistent with LS-associated rectal cancer.

## Treatment

Given the tumor size and late-stage presentation, multidisciplinary team (MDT) evaluation involving colorectal surgery, oncology, obstetrics, and genetics was performed. MDT considered standard neoadjuvant chemoradiotherapy, but the patient’s mid-pregnancy status posed high fetal risk. Formal genetic counseling was provided during MDT discussions, addressing the diagnosis of Lynch syndrome, the autosomal dominant inheritance pattern, and the associated 50% risk of transmission to offspring. The potential reproductive and ethical implications were incorporated into the overall clinical decision-making process. Considering the potential teratogenic risks associated with radiotherapy and cytotoxic chemotherapy during pregnancy, as well as the poor fetal viability at 22 weeks and the genetic implications of Lynch syndrome, medical termination of pregnancy was discussed within the multidisciplinary team. Following comprehensive counseling regarding maternal oncologic prognosis, fetal viability, and genetic risk, shared decision-making was undertaken, and the patient expressed full understanding of the clinical situation and consented to the recommended treatment strategy. Medical termination of pregnancy was subsequently performed under close monitoring with mifepristone and misoprostol, followed by curettage.

After recovery, the patient received neoadjuvant chemoradiotherapy (50.4 Gy/28 fractions) combined with two cycles of capecitabine plus oxaliplatin (CapeOx) plus tislelizumab (oxaliplatin 200 mg IV day 1; capecitabine 1500 mg orally twice daily, days 1–14; tislelizumab 200 mg IV day 1; every 3 weeks). Post-treatment reassessment with CT and MRI showed tumor regression to yT2N0M0, and colonoscopy revealed a 3 × 3 cm scar-like lesion. Serum markers improved: CEA 2.2 ng/mL, CA 19-9 89.6 U/mL, AFP 4.64 ng/mL.

**Figure 4:**
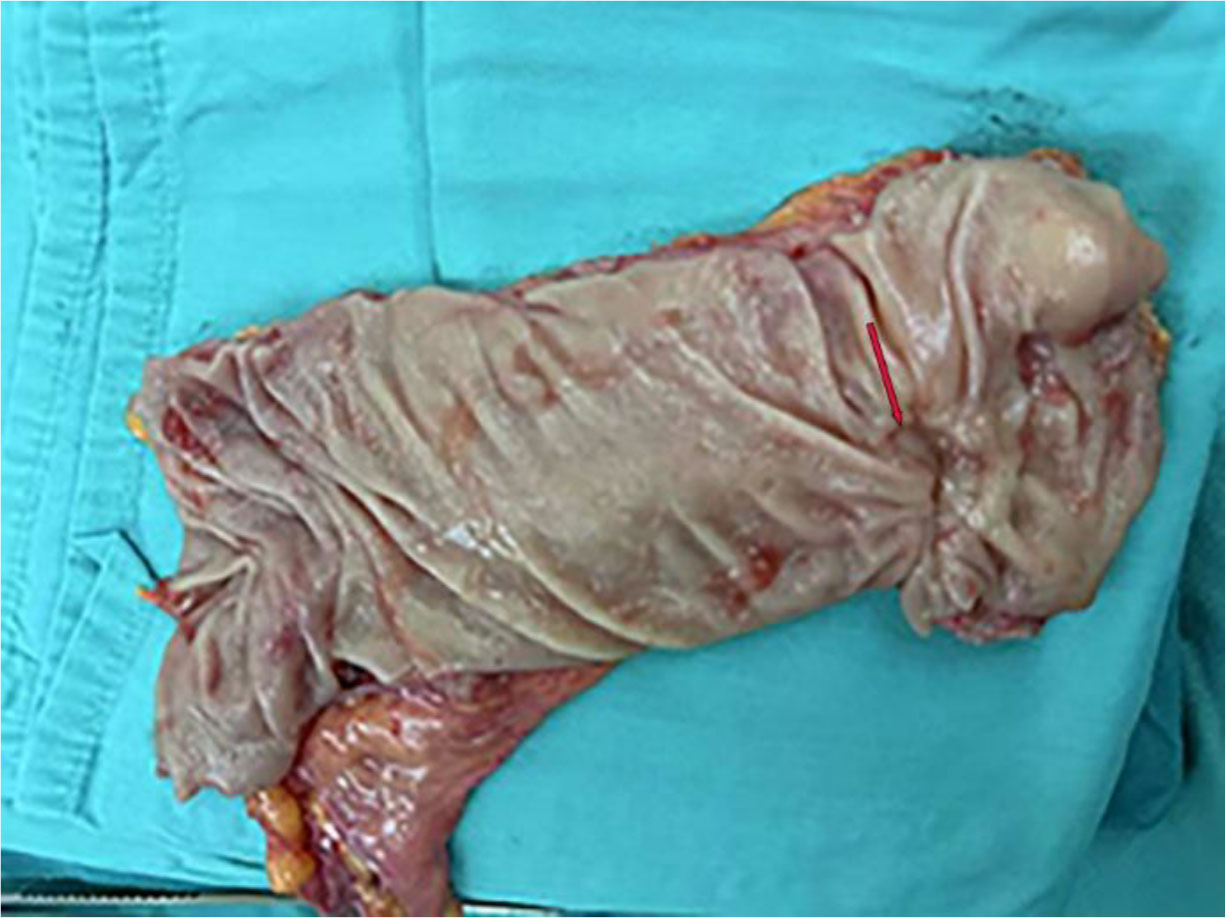
Gross specimen of the rectal tumor. Intraoperative specimen showing an ulcerative lesion measuring approximately 2.5 × 1.5 × 1.0 cm on the rectal wall.

Eight weeks after radiotherapy, laparoscopic Dixon resection was performed. Intraoperative gross specimen revealed a 2.5 × 1.5 × 1.0 cm ulcerative lesion (**Figure 4**). Pathology confirmed moderately differentiated adenocarcinoma, tumor regression grade 1 (TRG 1), ypT2N0M0. Postoperative adjuvant therapy included six cycles of CapeOx plus tislelizumab. Adverse effects during therapy were limited to grade 1–2 (CTCAE v5.0) nausea, anorexia, leukopenia, thrombocytopenia, and mild hepatic enzyme elevation, all resolving with supportive care. At 24-month follow-up, the patient remains disease-free. Given that the patient had just completed comprehensive oncologic treatment, she did not have immediate reproductive plans at the time of follow-up. However, she was informed about available reproductive options for future pregnancies, including preimplantation genetic testing for monogenic disorders (PGT-M), as a strategy to prevent vertical transmission of the pathogenic variant.

## Discussion

Pregnancy-associated colorectal cancer (pCRC) and Lynch syndrome (LS) are both rare, and their co-occurrence presents substantial diagnostic and therapeutic challenges. LS is an autosomal dominant hereditary cancer predisposition syndrome caused by germline mutations in mismatch repair genes (e.g., MLH1, MSH2, MSH6, PMS2) (1), conferring a lifetime colorectal cancer risk of 40–80% (2).

Several reports have described colorectal cancer diagnosed during pregnancy; however, most cases are sporadic and not associated with hereditary cancer syndromes, and standardized management remains undefined due to its rarity (3, 4). In contrast, colorectal cancer during pregnancy in patients with Lynch syndrome is extremely rare, with only very limited evidence reported in the literature (5). Moreover, current literature mainly focuses on diagnostic delay and surgical timing, while evidence regarding comprehensive multidisciplinary management incorporating neoadjuvant chemoradiotherapy and immunotherapy during pregnancy remains extremely limited.

Compared with previously reported cases, this case is characterized by the coexistence of Lynch syndrome and locally advanced rectal cancer during mid-pregnancy, which increases both oncologic and genetic complexity. Management was guided by a multidisciplinary team, leading to termination of pregnancy followed by neoadjuvant chemoradiotherapy combined with immunotherapy. The patient achieved a favorable pathological response (TRG 1) and remained disease-free at 24 months, suggesting that this approach may be feasible in selected patients.

Although LS is associated with various malignancies, reports of pCRC in LS patients are extremely limited, likely due to the low incidence of colorectal cancer during pregnancy (~0.002%) and the rarity of LS manifestation in this population (3). Early diagnosis is often delayed because pregnancy-related gastrointestinal symptoms can mask tumor-related signs, resulting in late-stage detection (4). Therefore, colorectal cancer occurring during pregnancy in patients with Lynch syndrome remains rare, and its diagnosis and management are particularly challenging. Because the clinical manifestations often resemble normal physiological changes of pregnancy, early diagnosis is frequently delayed, resulting in detection at more advanced stages of the disease (3). In addition, patients with Lynch syndrome during pregnancy face the risk of transmitting hereditary diseases, and pregnancy itself may influence the biological behavior of tumors (6). Therefore, tumor patients with Lynch syndrome during pregnancy require comprehensive evaluation and individualized management by a multidisciplinary team (7).

In patients with Lynch syndrome, due to its autosomal dominant inheritance pattern, each pregnancy carries a 50% risk that the offspring will inherit the pathogenic gene. The lifetime cumulative risk of developing colorectal cancer is approximately 66.08% in men and 42.71% in women, while women also face a 39.39% lifetime risk of endometrial cancer (8). Since there are currently no curative preventive measures and periodic screening or follow-up cannot eliminate the underlying genetic risk, reproductive genetic intervention has become a crucial consideration for carriers planning to conceive. Preimplantation genetic testing for monogenic disorders (PGT-M) has emerged as a feasible approach to prevent the transmission of Lynch syndrome–related pathogenic variants. This technique involves obtaining embryos through *in vitro* fertilization (IVF), performing genetic testing on a small number of cells from the blastocyst stage, and selecting embryos free of the familial pathogenic mutation for transfer, thereby effectively preventing transmission of the disease-causing gene. Several studies have demonstrated that PGT-M is accurate and feasible for Lynch syndrome and other hereditary cancer syndromes, providing a means of fundamental prevention at the reproductive level (9). For patients who do not undergo PGT-M, prenatal genetic testing—such as chorionic villus sampling or amniocentesis—can still be performed during pregnancy to determine the fetal genotype. The results can then guide pregnancy management decisions following comprehensive genetic counseling.

At 22 weeks of gestation, the likelihood of fetal survival outside the uterus remains extremely low. Studies have shown that the survival rate of infants born at 22 weeks is only about 6–10%, while it increases to approximately 43.2% at 25 weeks. Even under advanced neonatal intensive care conditions, survivors are often affected by severe neurodevelopmental complications, including cerebral palsy, visual and auditory impairment, and cognitive disability (10, 11). Therefore, in cases where the mother requires prompt oncologic treatment, delaying induction of labor to allow further fetal maturation carries substantial risk.

For locally advanced rectal cancer (LARC), neoadjuvant therapy plays a key role in improving local control and reducing the risk of distant metastasis. Conventional neoadjuvant strategies include radiotherapy or chemoradiotherapy (CRT), which aim to shrink tumor size, increase the rate of curative resection, and reduce local recurrence. In the context of pregnancy-associated rectal cancer, chemotherapy options such as oxaliplatin and capecitabine pose significant reproductive toxicity. Oxaliplatin has been associated with fetal developmental abnormalities, bone marrow suppression, and neurotoxicity, while capecitabine can cross the placenta and interfere with fetal DNA synthesis, increasing the risk of miscarriage, intrauterine fetal demise, and severe congenital malformations (12, 13). In recent years, the use of immune checkpoint inhibitors (PD-1/PD-L1 inhibitors) in neoadjuvant therapy has gained increasing attention. Early studies suggest that combining immunotherapy with standard chemoradiotherapy can enhance antitumor immune responses by promoting tumor-infiltrating lymphocyte (TIL) accumulation, thereby improving the rate of pathological complete response (pCR) before surgery (14). Furthermore, neoadjuvant immunotherapy combinations have shown superior efficacy in a subset of rectal cancers characterized by high microsatellite instability (MSI-H) or mismatch repair deficiency (dMMR), highlighting the potential of precision immunotherapy as a future direction in rectal cancer management. However, for pregnant patients requiring anticancer treatment, particularly those receiving immune checkpoint inhibitors (PD-1/PD-L1 blockade), animal studies have demonstrated that these agents can impair placental function and lead to miscarriage, stillbirth, or fetal growth restriction (15). Existing human case reports also suggest increased risks of preterm birth and adverse fetal outcomes (16). Given the limited clinical data, the use of immune checkpoint inhibitors during pregnancy is generally not recommended, and effective contraception along with multidisciplinary risk assessment should be emphasized for women of reproductive age.

Radiotherapy during pregnancy also carries significant risks, which depend mainly on the radiation dose, gestational age, and the treatment field. During early pregnancy, particularly in the period of organogenesis, the fetus is highly sensitive to radiation exposure; high doses can result in miscarriage or severe congenital malformations (17). Pelvic radiotherapy for rectal cancer inevitably involves direct fetal exposure, even when advanced techniques such as intensity-modulated radiotherapy (IMRT/VMAT) or image-guided radiotherapy (IGRT) are used. Dosimetric studies have shown that when the target dose is approximately 20 Gy, the estimated scattered dose to the uterus is about 9–16 mGy in early pregnancy and may rise to 13–20 mGy in later stages. Although these doses are far below the lethal threshold, radiation exposure may still affect rapidly dividing fetal cells, potentially increasing the risk of DNA damage, organ developmental abnormalities, and long-term neurocognitive impairment (18). Classical reviews have also indicated that high-dose abdominal or pelvic irradiation—particularly when the uterus is directly or closely exposed—is associated with an increased risk of miscarriage, low birth weight, intrauterine growth restriction (IUGR), and preterm delivery (19). Moreover, although large-scale human data remain limited, recent follow-up studies of children exposed to radiotherapy *in utero* have generally shown normal average neurocognitive, psychosocial, and physical development outcomes, while isolated cases suggest the possibility of subtle functional impairments (20).

Therefore, for pregnant patients with cancer, systemic chemotherapy, radiotherapy, or immunotherapy is generally not recommended unless maternal life is at immediate risk. In clinical practice, multidisciplinary teams carefully weigh maternal benefit against fetal risk and often prefer to postpone treatment until after delivery, or, when medically appropriate, to initiate definitive oncologic therapy immediately following induction of labor. This approach balances the need for effective maternal treatment with fetal safety considerations. Given the extremely low likelihood of fetal survival under aggressive oncologic treatment and the potential teratogenic effects of chemotherapy, medically indicated termination of pregnancy may be considered a reasonable strategy to enable timely initiation of standard therapy. Such decisions are typically made through multidisciplinary consultation involving obstetrics, oncology, genetics, and neonatology to ensure a balanced assessment of maternal safety, treatment timing, and fetal risk.

For women of reproductive age with hereditary cancer syndromes such as Lynch syndrome who wish to conceive, preimplantation genetic testing for monogenic disorders (PGT-M) or prenatal genetic screening can be pursued under professional genetic counseling and ethical guidance. These interventions effectively prevent transmission of pathogenic variants to offspring and reduce cancer susceptibility in the next generation, providing a medically sound and ethically responsible reproductive option for high-risk families.

## Conclusion

Locally advanced rectal cancer in pregnant LS patients is exceedingly rare and requires a careful balance between maternal tumor control, fetal safety, and genetic considerations. In mid-gestation, due to low fetal viability and high treatment-related teratogenic risk, medical termination may be justified to enable timely neoadjuvant chemoradiotherapy and immunotherapy. Individualized, multidisciplinary management strategies can optimize maternal outcomes while scientifically mitigating fetal and genetic risks, providing a feasible approach for high-risk pregnancy-associated malignancies.

## Data Availability

The original contributions presented in the study are included in the article/supplementary material. Further inquiries can be directed to the corresponding author.
